# Perforation of the Membrana Tympani for Cure of Deafness

**Published:** 1846-08

**Authors:** 


*Perforation of the Membrana Tympani for the Cure of Deafness.*—A young woman was completely deaf in both ears for four years, caused by a severe cold. Catheterism of the Eustachian tube was performed, and said to fail. The membrane of the tympanum was pierced, a small piece being drilled out of the membrane of the right side. After the operation, the hearing was greatly improved. Next day, intense pain was experiencad in the ear. Remedies were applied, and in forty-eight hours a profuse discharge took place from the ear. This varied in quantity, day after day, for two months, the hearing being slightly benefited. In eleven weeks after the operation, the patient became more deaf than ever, and was constantly complaining of flying pains through the head; at one time fixing in the forehead for a few hours, at another in the occiput, and frequently in the temple, particularly in the right ear. In this state she continued for four months, when she was attacked with rigors, rapid pulse, intolerance of light, and all the symptoms of disease in the brain, and she died on the third day.

On examination, an abscess was found in the lower part of the middle lobe of the brain. No opening could be detected through the petrous portion of the temporal bone, but the dura matter covering it was roughened on the surface, and softened in its texture, particularly near the internal auditory foramen. The membrana tympani was entirely destroyed, and the lining membrane of the tympanum was considerably thickened and villous on its surface.—*Dublin Medical Press.—Boston Medical and Surgical Journal.*

